# Computational and experimental methods to decipher the epigenetic code

**DOI:** 10.3389/fgene.2014.00335

**Published:** 2014-09-23

**Authors:** Stefano de Pretis, Mattia Pelizzola

**Affiliations:** Computational Epigenomics, Center for Genomic Science of IIT@SEMM, Fondazione Istituto Italiano di TecnologiaMilan, Italy

**Keywords:** epigenetic code, histone code, chromatin state, histone mark, epigenome editing, chromatin regulators

## Abstract

A multi-layered set of epigenetic marks, including post-translational modifications of histones and methylation of DNA, is finely tuned to define the epigenetic state of chromatin in any given cell type under specific conditions. Recently, the knowledge about the combinations of epigenetic marks occurring in the genome of different cell types under various conditions is rapidly increasing. Computational methods were developed for the identification of these states, unraveling the combinatorial nature of epigenetic marks and their association to genomic functional elements and transcriptional states. Nevertheless, the precise rules defining the interplay between all these marks remain poorly characterized. In this perspective we review the current state of this research field, illustrating the power and the limitations of current approaches. Finally, we sketch future avenues of research illustrating how the adoption of specific experimental designs coupled with available experimental approaches could be critical for a significant progress in this area.

## INTRODUCTION

Histone post-translational modifications (PTMs; Kouzarides, 2007) and DNA methylation (Pelizzola and Ecker, 2011) are the main constituent of the epigenome, which greatly contributes to the definition of cells’ identity through the instruction of specific transcriptional and regulatory programs (Rivera and Ren, 2013). Histone PTMs mostly occur on the amino-terminal tails of the core histone proteins, which protrude through the DNA backbone and are exposed on the nucleosomal surface where they are subjected to a wide range of enzyme-catalyzed modifications (Rothbart and Strahl, 2014). These include acetylation of lysine, methylation of both lysine and arginine and phosphorylation of serine, and threonine residues. It is also possible to distinguish these PTMs based on the number of such modifications (e.g., mono-, di-, or tri-methylation of lysines), and based on the symmetry (or lack of) of the modification over the two copies of each core histone (Voigt et al., 2012). Histone PTMs are recognized by specific binding proteins, acting as docking point for chromatin regulators (CRs), which in turn could trigger further modifications in a chain process (Schreiber and Bernstein, 2002).

Assigning a clear and distinct functional role to each histone PTM has proven to be elusive. For example, phosphorylation of H3 at serine 10 is associated with both chromosome condensation during mitosis (Hendzel et al., 1997; Wei et al., 1999) and transcriptional activation following mitogenic stimulation (Barratt et al., 1994). Similarly, histone acetylation, which is commonly associated to active chromatin, can also be linked to gene repression (Braunstein et al., 1996; De Rubertis et al., 1996). Moreover, DNA methylation can be an important feature in either repressed or actively transcribed genes, depending on the localization at the level of promoter or gene-body, respectively (Pelizzola and Ecker, 2011; Baubec and Schübeler, 2014).

These observations led to the formulation of the histone code hypothesis (Strahl and Allis, 2000) which, including DNA methylation, can be extended to the more general epigenetic code. The main idea behind the histone code is that the functional role of histone PTMs is better defined taking into account multiple histone modifications acting in a combinatorial or sequential fashion, specifying unique downstream functions. The compliance of the histone code to the semiotic definition of a code was discussed by Turner (). In that context, a code is a system made of signs, to which a meaning is assigned by the rules of the code itself. The rules, in biology are the “readers” of the code: for the genetic code tRNAs, for the epigenetic code CRs. Following the semiotic definition, no causal relationships should exist between the sign and its meaning. However, as in the cases of lysine acetylation and serine phosphorylation, some modifications, or signs, physically predispose the local environment to the fulfillment of their meaning. Probably many real codes break semiotic rules in order to reduce reading errors and increase robustness. For example, in the traffic-light code it was decided to rely on red, a color associated with fear, to convey the action of stopping. A good code is a code that works, at the end.

The existence of the epigenetic code is supported by the observation that CRs are often more specific for peptides marked by multiple modifications (Morinière et al., 2009; Ruthenburg et al., 2011). Additionally, CRs can be multimeric complexes containing multiple recognition sites (Lindroth et al., 2004). Genome-wide mapping studies found limited combinatorial complexity of the marks (Huff et al., 2010; Ernst et al., 2011) compared to the number of theoretically possible combinations, but in our opinion, this does not represent an argument against the existence of the epigenetic code. Nevertheless, the emergence of the histone code hypothesis was followed by a series of criticisms. First, Rando (2012) commented that the histone code hypothesis does not provide any additional insight into the reason why a given combination of histone modifications, that globally co-occur, only affects a small subset of genes. We note here that typically only a subset of the known marks is actually profiled and that the complete set of modifications has likely not been identified yet. Thus, it is conceivable that the inclusion of the missing marks could shed light on those discrepancies. Second, limited complexity at the level of the transcriptional response is associated to mutations of lysines in the N-terminal tails of core histones in yeast (Martin et al., 2004). This could either suggest relevant redundancy in the functional role of these PTMs, or the fact that these mutations could result in other types of phenotypic responses. Finally, the transcriptional consequences of combinations of histone mutations affecting their PTMs could be in some cases predicted by the linear combination of individual mutations, suggesting that some of these modification could be read separately (Dion et al., 2005; Yuan et al., 2006). Eventually, despite debate about the complexity and prevalence of this code, this is an active area of research in both experimental and computational genomics.

## COMPUTATIONAL METHODS FOR THE IDENTIFICATION OF CHROMATIN STATES

Following the histone code hypothesis, computational tools were developed for the identification of recurrent combinations of histone PTMs. Combinations associated to transcribed regions, active promoters, and enhancers were recognized and used to identify new occurrences of the same regions, allowing the prediction of new non-coding transcripts (Guttman et al., 2009; Fatica and Bozzoni, 2013) and enhancers sites (Heintzman et al., 2007; Wang et al., 2013).

**FIGURE 1:**
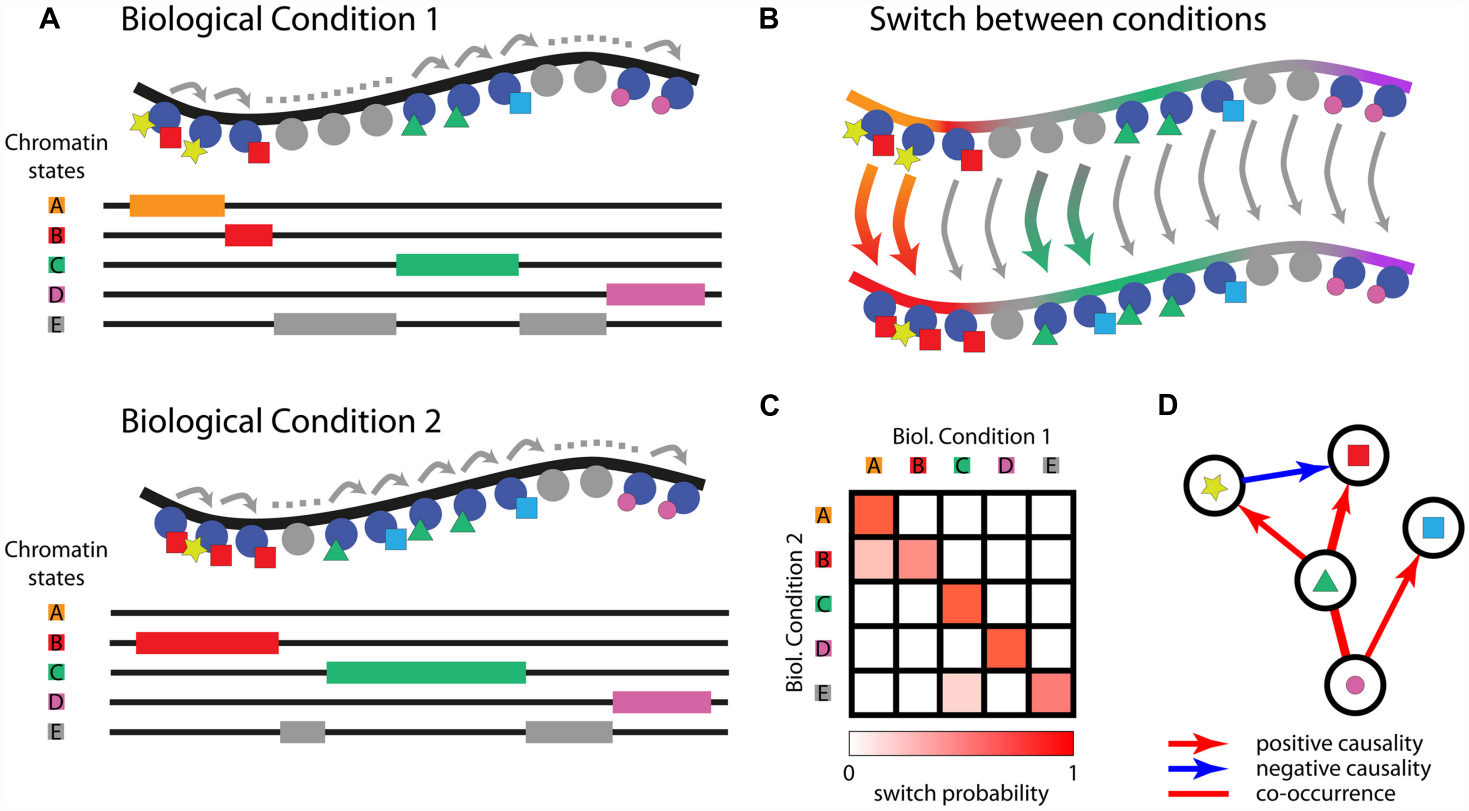
**Adopting chromatin states to decipher the interplay between epigenetic marks across multiple biological conditions.** HMM-based learning of chromatin states; DNA is depicted in black, histones as blue or gray circles, and different histone’s PTMs as colored shapes. Chromatin states identifying relevant combinations of histones PTMs are drawn in the underlying diagram **(A)**. Chromatin states can be compared over different cell types or biological conditions; arrows represent the switch between different states **(B)**. Heatmap displaying the probability of switching between chromatin states in different biological conditions **(C)**. Graph depicting causal relationships among epigenetic marks determined based on **(C,D)**.

Conversely, computational tools were developed for the unsupervised identification of recurrent combinations of histone PTMs, showing that the chromatin can be described by a limited number of chromatin states that are specifically enriched in functional genomic regions. The most widely used unsupervised tools are ChromaSig (Hon et al., 2008) and ChromHMM (Ernst and Kellis, 2012). ChromaSig was applied for the identification of 16 clusters of histone modifications using genome-wide maps of 21 chromatin marks from ChIP-chip experiments in HeLa cells. ChromHMM was applied for the analysis of 38 histone modifications, H2AZ, RNA polymerase II, and CTCF in human CD4 T-cells and allowed the identification of 51 distinct chromatin states using multivariate Hidden Markov Models. These states could be subsequently matched to functional genomic elements and distinguished into six broad classes of chromatin states: promoter, enhancer, insulator, transcribed, repressed, and inactive states. ChromHMM is also able to classify chromatin in regions strongly depleted of histone modifications. Overall, the results of the two methods applied to the same dataset are highly correlated (Ernst and Kellis, 2010). These tools are currently the reference for a holistic view of the chromatin and for associating complex combinatorial signatures of histone PTMs with critical functional elements of the genome (**Figure 1A**).

It is now clear that out of the enormous theoretical combinatorial complexity of histone PTMs, only a subset of these combinations seem to occur in nature. Histone modifications are highly related to each other, some of them are highly co-occurring while other are clearly anti-correlated, greatly reducing the combinatorial complexity. Taking a step ahead in the study of the correlation structure among these marks, a couple of studies was recently published adopting approaches based on (i) partial correlations and (ii) maximum entropy modeling. On one hand, partial correlations were used to discriminate the cases where two modifications are both strongly related to a third one, prompting the possibility that their correlation is only an indirect effect. Partial correlation between marks A and B is determined as the correlation of the residuals arising from the linear modeling of the individual marks (A and B) with an additional factor (mark C). If the residuals are not correlated it means that the correlation between A and B is likely due to the similarity to the mark C (Lasserre et al., 2013). On the other hand, a framework based on maximum entropy modeling was adopted to decipher pairwise and higher-order interactions between chromatin factors (Zhou and Troyanskaya, 2014). Approaches like these are critical to obtain more meaningful network relating different marks, disentangling direct from indirect similarities.

## DYNAMICS OF CHROMATIN STATES

While important progress was recently made to increase the likelihood of identifying biologically relevant similarities and interactions among epigenetic marks (Lasserre et al., 2013; Zhou and Troyanskaya, 2014), new computational tools and carefully designed experiments are needed to progress from correlative to causal analyses. Most of the studies in this area are conducted using data collected under static conditions, or steady state, and are limited to the identification of networks of epigenetic marks that are necessarily undirected and lack causality. Indeed, after the formalization of the histone code hypothesis, numerous experimental and computational efforts have been carried over to chart chromatin states in a plethora of biological conditions and cell types. These efforts raised the consciousness that similar chromatin states exist in different cell types but they are in some cases displaced (Ernst et al., 2011; Ernst and Kellis, 2013). To this purpose, a non-parametric method for the analysis of differential chromatin modifications (dCMD) was developed for the identification of cell-type specific regulatory elements (Chen et al., 2013), based on the same data used in (Ernst et al., 2011). The comprehension on the mechanism of this displacement, which leads specific portions of the chromatin to change their configuration and possibly the expression pattern, requires following these processes step by step. It is now time for a better understanding on the mechanisms that are responsible for the establishment of a given chromatin state and for its subsequent modification. Given a chromatin state composed of marks A, B, and C in a biological condition X, is there a specific order in their deposition that determined the emergence of the combinatorial pattern as a stable and relevant state? Given a biological condition Y derived from X, which mechanisms determine the establishment of differential patterns of chromatin states (**Figure 1B**)? Using Bayesian statistics, recent works tried to infer causality among different marks using only data from single steady state conditions (Yu et al., 2008). We believe that this task would be greatly facilitated by the study of chromatin state dynamics in subsequent biological conditions, such as in the course of consecutive differentiation stages or in general following biological responses over time. This would be critical to dissect which histone marks are more important in positioning other marks in subsequent conditions (**Figures 1C,D**). While highly relevant, efforts made to explore the variation of chromatin states between different cell types (Ernst et al., 2011) are limited in terms of improving our comprehension about the mechanisms that could have brought to those alternative differentiation end points. Multiple differentiation steps could have been missed, which could be critical for comprehending the mechanisms bringing from one chromatin state to the following. Choosing the right experimental design, or conditions to compare, is not straightforward. Even comparing consecutive differentiation stages can be uninformative, because not every differentiation step involves chromatin remodeling. During erythroid differentiation, for example, chromatin states are established at the stage of lineage commitment and extensive changes in gene expression follow a different recruitment of the master transcription factor GATA1, while the chromatin state profiles and accessibility remain largely unchanged (Wu et al., 2011).

## EPIGENETIC CODE: THE ROLE OF THE CHROMATIN REGULATORS

As previously noted, a code is made essentially by three components: the sign, the meaning, and the reader of the sign (Turner, 2007). According to that, the same sign could convey a different meaning given different readers, meaning that the expression of a different set of CRs could determine an alternative readout of the same epigenetic marks, while leaving the signs (the marks) unchanged. As discussed above, the same histone modifications could have different functional roles in different moments of the cell cycle, as in the case of the H3S10 phosphorylation during mitosis and mitogenic stimulation (Strahl and Allis, 2000). The two most likely explanations are that additional modifications have to be taken into account to confer the right meaning to that shared sign (mark), or that there is some difference at the level of the readers. Strahl and Allis (2000) in their pioneer article on the histone code, hypothesized that “part of the solution to this paradox may be in having unique histone codes read by distinct sets of proteins that then bring about different downstream responses. If correct, it may be that mitosis-specific HATs, HDACs, and HMTs act during chromosome condensation and that distinct sets of histone-modifying enzymes mark chromatin for decondensation during gene activation” (Strahl and Allis, 2000). Indeed different cell types have been described to express different sets of CRs (Ho and Crabtree, 2010) among the 100s encoded by the human genome (Kouzarides, 2007; Ruthenburg et al., 2007), leading to a possible context dependent interpretation of the marks. During development, for example, different combinations of the vertebrate SWI/SNF complexes undergo progressive changes in subunit composition, from pluripotent stem cells to multipotent neuronal progenitor cells to a committed neuron (Lessard et al., 2007; Yan et al., 2008). Alterations in the composition of these complexes at the level of specific subunits can influence the ability of these cell types to self-renew or differentiate (Lessard et al., 2007; Ho et al., 2009), suggesting that the cell specific reader is critical to determine cell identity. CRs have been identified as a new set of driver genes in different types of tumors (Elsässer et al., 2011) confirming their relevance.

Chromatin regulators not only need to be correctly expressed in the specific cell type, but also need gain access to the locus where the target histone mark is located in order to exploit their function. In a recent study, chromatin localization of 29 CRs was profiled genome-wide in K562 cells and human ESCs (Ram et al., 2011). Very recently, a powerful experimental methodology was developed to study the substrate specificity of CRs, based on the semi-synthesis of nucleosome libraries with distinct combinations of PTMs (Nguyen et al., 2014). Similar approaches can help elucidating the interaction between chromatin states and CRs, being able to properly assign distinct functions to each of these ensembles.

Turner (2007) suggested the following definition for the epigenetic code: it “describes the way in which the potential for expression of genes in a particular cell type is specified by chromatin modifications put in place at an earlier stage of differentiation.” This definition particularly fits in the context described above, in which the presence of a specific reader could eventually put into effect the meaning of a previously set epigenetic mark.

## EXPERIMENTAL METHODS FOR INVESTIGATING THE EPIGENETIC CODE

The success in deciphering the epigenetic code depends on the availability of new computational methods and the generation of suitable datasets. The latter have to be as informative as possible on the combinatorial combinations in which the epigenetic marks could occur. The highest is the coverage of any possible occurring combination, the highest is the likelihood for the computational tools to capture it. Two types of datasets are most suited for these analyses: (i) large-scale public datasets, and (ii) *ad hoc* perturbation datasets.

**FIGURE 2:**
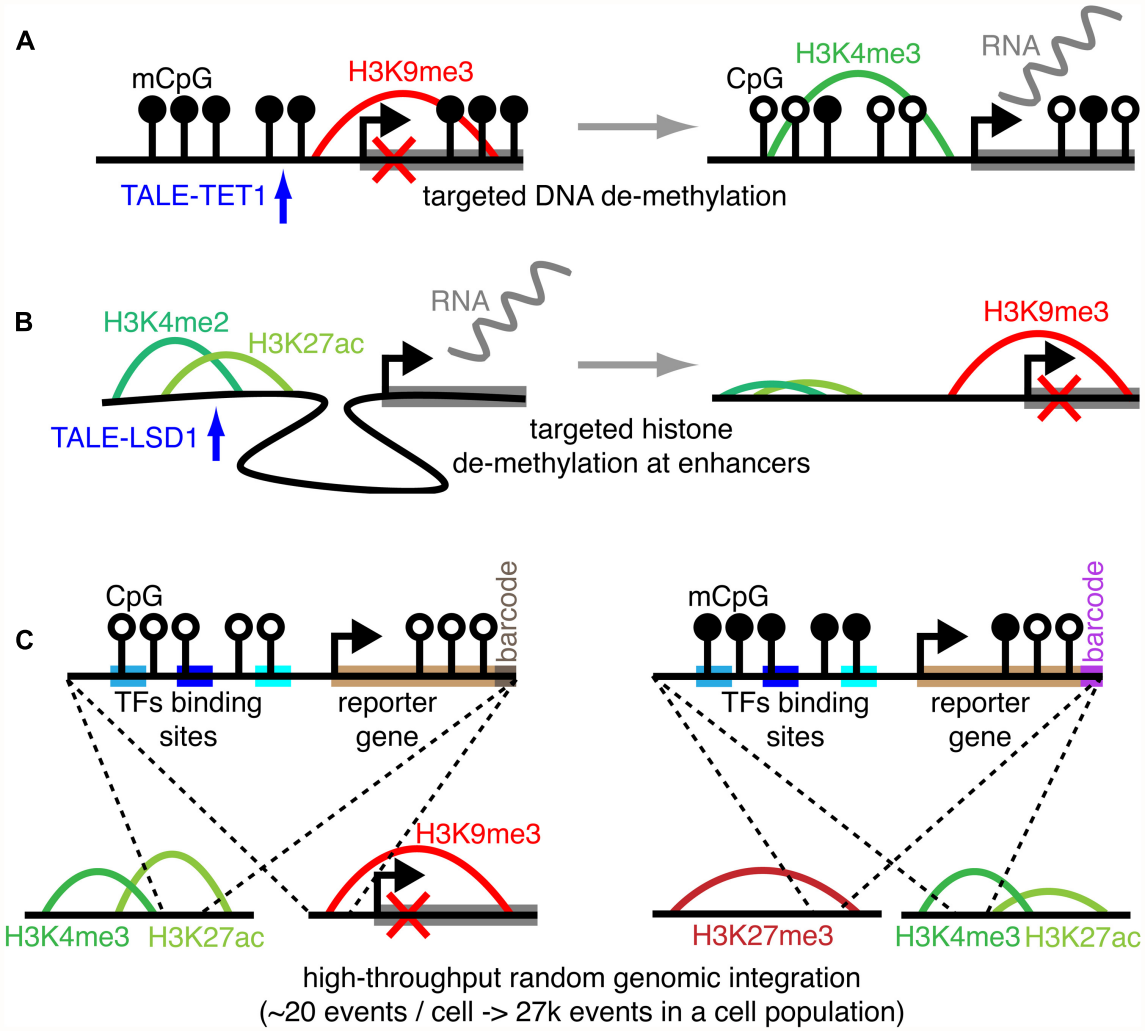
**Experimental methods to probe the epigenetic code.** Using a TALE-TET1 construct to determine TET1-mediated DNA de-methylation of a specific promoter, resulting in the transcriptional induction of the downstream gene **(A)**. Using a TALE-LSD1 construct to determine LSD1-mediated histone de-methylation of a specific active enhancer, resulting in enhancer inactivation and the transcriptional repression of the target gene **(B)**. Simplified representation of the thousands of reporters integrated in parallel (TRIP) method that can be used to probe the epigenome with reporter genes set under control of specific regulatory and epigenetic input, here depicted as transcription factor (TFs) binding sites and DNA methylation **(C)**.

Large-scale datasets such as ChIP-chip and ChIP-seq samples stored in public repositories currently account for 1000s of samples in various species, tissues, and conditions, representing a formidable resource covering numerous epigenetic combinatorial states. Still, while numerous, these datasets do not guarantee complete covering of all possible combinations among known epigenetic marks. To overcome this issue, it is nowadays possible to take advantage of experimental methods to build perturbation experiments, providing medium- or high-throughput datasets where the combinations among epigenetic marks are explored through direct perturbation of a baseline combinatorial state. These experiments naturally provide important hints on the causal mechanisms determining the occurrence of these combinatorial states. Among these experimental procedures (i) precision epigenetic engineering and (ii) high-throughput screening methods are emerging as powerful tools to rewire the epigenome. Examples of the former are epigenome-editing tools such as transcription activator-like effector repeat arrays (TALE) and Zinc fingers, which can be coupled with enzymes modifying a given epigenetic mark in targeted genomic regions. Specifically, engineered TALE were fused to the TET1 hydroxylase catalytic domain to obtain targeted demethylation of endogenous promoters in human cells, determining increased transcription of the downstream genes (Maeder et al., 2013). Similarly, another group was able to fuse TALE with the LSD1 histone de-methylase to remove H3K4me3 at the level of enhancer sites, driving transcriptional repression of the target gene (Mendenhall et al., 2013; **Figures 2A,B**). Using zinc finger proteins (ZFPs), 223 CRs were fused to ZFPs in a remarkable effort to study the transcriptional logic resulting from combinatorial recruitment of CRs in yeast (Keung et al., 2014). Finally, coupling of the DNMT3a catalytic domain with ZFPs allowed targeted methylation of the promoter of the tumor suppressor *Maspin* determining its stable transcriptional repression over multiple cell generations, even in absence of sustained presence of the ZFPs (Rivenbark et al., 2012). Ongoing research is focused on the development of similar tools adopting a more flexible methodology based on clustered regularly interspaced short palindromic repeats (CRISPR). CRISPR constructs associated to catalytic domains of enzymes targeting the epigenome could conveniently be directed to specific genomic loci through the simple transfection of guide-RNA molecules complementary to the target region.

Importantly these methods are suitable for multiplexing, allowing in principle to target various enzymatic activities of interest in multiple genomic regions. These methods are still under development, including a better quantification and minimization of off-target effects. Nevertheless, published proof of concept experiments suggest that these tools offer a unique opportunity for interfering with the epigenetic code, injecting controlled, and targeted epigenetic alterations and opening new avenues of research in rewiring epigenome code in normal and cancer cells (Blancafort et al., 2013). Finally, matching these epigenetic perturbations with the consequent alteration in the binding of regulatory proteins and gene transcriptional activity will provide data suitable for reverse engineering the interplay among epigenetic, regulatory, and transcriptional layers.

On the other hand, high-throughput screening methods were developed to probe the epigenome by measuring how the local epigenetic state influences the expression of reporter genes randomly integrated in the genome. In particular, the thousands of reporters integrated in parallel (TRIP) method was recently developed to target 1000s of random genomic loci in a cell population with a gene reporter. The promoters controlling the reporter expression can be designed to contain specific transcriptional factor binding sites and/or to host methylated cytosines (**Figure 2C**). This would allow us to associate the reporter gene activity to both a specific regulatory/epigenetic input and the epigenetic state of the insertion region. Indeed, both the location of the insertion events and the normalized expression of the reporter gene can be determined through simple high-throughput sequencing of barcoded sequences, without cloning, greatly simplifying the experimental setting and accelerating the acquisition of the data (Akhtar et al., 2013, 2014).

## CONCLUSION

The comprehension on the mechanism regulating the intricate networks of epigenetic modifications represents a formidable while exciting area of research. Significant progress is currently being made thanks to the advent of high-throughput sequencing technologies, which are allowing an unprecedented accumulation of data and the development of computational tools developed to characterize the combinatorial nature of the epigenome. It is now time to design experiments which are directly intended to challenge and deepen our knowledge in this field, taking advantage of powerful epigenome-editing and high-throughput screening experimental methodologies which are currently being made available.

## Conflict of Interest Statement

The authors declare that the research was conducted in the absence of any commercial or financial relationships that could be construed as a potential conflict of interest.

## References

[B1] Akhtar W. de Jong J. Pindyurin A. V. Pagie L. Meuleman W. de Ridder J. (2013). Chromatin position effects assayed by thousands of reporters integrated in parallel. *Cell* 154 914–927 10.1016/j.cell.2013.07.018 23953119

[B2] Akhtar W. Pindyurin A. V. de Jong J. Pagie L. Hoeve ten J. Berns A. (2014). Using TRIP for genome-wide position effect analysis in cultured cells. *Nat. Protoc.* 9 1255–1281 10.1038/nprot.2014.072 24810036

[B3] Barratt M. J. Hazzalin C. A. Cano E. Mahadevan L. C. (1994). Mitogen-stimulated phosphorylation of histone H3 is targeted to a small hyperacetylation-sensitive fraction. *Proc. Natl. Acad. Sci. U.S.A.* 91 4781–4785 10.1073/pnas.91.11.4781 8197135 PMC43872

[B4] Baubec T. Schübeler D. (2014). Genomic patterns and context specific interpretation of DNA methylation. *Curr. Opin. Genet. Dev.* 25 85–92 10.1016/j.gde.2013.11.015 24614011

[B5] Blancafort P. Jin J. Frye S. (2013). Writing and rewriting the epigenetic code of cancer cells: from engineered proteins to small molecules. *Mol. Pharmacol.* 83 563–576 10.1124/mol.112.080697 23150486 PMC3920093

[B6] Braunstein M. Sobel R. E. Allis C. D. Turner B. M. Broach J. R. (1996). Efficient transcriptional silencing in *Saccharomyces cerevisiae* requires a heterochromatin histone acetylation pattern. *Mol. Cell. Biol.* 16 434 9–4356 10.1128/mcb.16.8.4349 PMC231433 8754835

[B7] Chen C. Zhang S. Zhang X. S. (2013). Discovery of cell-type specific regulatory elements in the human genome using differential chromatin modification analysis. *Nucleic Acids Res.* 41 9230–9242 10.1093/nar/gkt712 23945931 PMC3814353

[B8] De Rubertis F. Kadosh D. Henchoz S. Pauli D. Reuter G. Struhl K. (1996). The histone deacetylase RPD3 counteracts genomic silencing in *Drosophila* and yeast. *Nature* 384 589–591 10.1038/384589a0 8955276

[B9] Dion M. F. Altschuler S. J. Wu L. F. Rando O. J. (2005). Genomic characterization reveals a simple histone H4 acetylation code. *Proc. Natl. Acad. Sci. U.S.A.* 102 5501–5506 10.1073/pnas.0500136102 15795371 PMC555684

[B10] Elsässer S. J. Allis C. D. Lewis P. W. (2011). Cancer. New epigenetic drivers of cancers. *Science* 331 1145–1146 10.1126/science.1203280 21385704

[B11] Ernst J. Kellis M. (2010). Discovery and characterization of chromatin states for systematic annotation of the human genome. *Nat. Biotechnol.* 28 817–825 10.1038/nbt.1662 20657582 PMC2919626

[B12] Ernst J. Kellis M. (2012). ChromHMM: automating chromatin-state discovery and characterization. *Nat. Methods* 9 215–216 10.1038/nmeth.1906 22373907 PMC3577932

[B13] Ernst J. Kellis M. (2013). Interplay between chromatin state, regulator binding, and regulatory motifs in six human cell types. *Genome Res.* 23 1142–1154 10.1101/gr.144840.112 23595227 PMC3698507

[B14] Ernst J. Kheradpour P. Mikkelsen T. S. Shoresh N. Ward L. D. Epstein C. B. (2011). Mapping and analysis of chromatin state dynamics in nine human cell types. *Nature* 473 43–49 10.1038/nature09906 21441907 PMC3088773

[B15] Fatica A. Bozzoni I. (2013). Long non-coding RNAs: new players in cell differentiation and development. *Nat. Rev. Genet.* 15 7–21 10.1038/nrg3606 24296535

[B16] Guttman M. Amit I. Garber M. French C. Lin M. F. Feldser D. (2009). Chromatin signature reveals over a thousand highly conserved large non-coding RNAs in mammals. *Nature* 457 223–227 10.1038/nature07672 19182780 PMC2754849

[B17] Heintzman N. D. Stuart R. K. Hon G. Fu Y. Ching C. W. Hawkins R. D. (2007). Distinct and predictive chromatin signatures of transcriptional promoters and enhancers in the human genome. *Nat. Genet.* 39 311–318 10.1038/ng1966 17277777

[B18] Hendzel M. J. Wei Y. Mancini M. A. Van Hooser A. Ranalli T. Brinkley B. R. (1997). Mitosis-specific phosphorylation of histone H3 initiates primarily within pericentromeric heterochromatin during G2 and spreads in an ordered fashion coincident with mitotic chromosome condensation. *Chromosoma* 106 348–360 10.1007/s004120050256 9362543

[B19] Ho L. Crabtree G. R. (2010). Chromatin remodelling during development. *Nature* 463 474–484 10.1038/nature08911 20110991 PMC3060774

[B20] Ho L. Ronan J. L. Wu J. Staahl B. T. Chen L. Kuo A. (2009). An embryonic stem cell chromatin remodeling complex, esBAF, is essential for embryonic stem cell self-renewal and pluripotency. *Proc. Natl. Acad. Sci. U.S.A.* 106 5181–5186 10.1073/pnas.0812889106 19279220 PMC2654396

[B21] Hon G. Ren B. Wang W. (2008). ChromaSig: a probabilistic approach to finding common chromatin signatures in the human genome. *PLoS Comput. Biol.* 4:e1000201 10.1371/journal.pcbi.1000201 PMC2556089 18927605

[B22] Huff J. T. Plocik A. M. Guthrie C. Yamamoto K. R. (2010). Reciprocal intronic and exonic histone modification regions in humans. *Nat. Struct. Mol. Biol.* 17 1495–1499 10.1038/nsmb.1924 21057525 PMC3057557

[B23] Keung A. J. Bashor C. J. Kiriakov S. Collins J. J. Khalil A. S. (2014). Using targeted chromatin regulators to engineer combinatorialand spatial transcriptional regulation. *Cell* 158 110–120 10.1016/j.cell.2014.04.047 24995982 PMC4110908

[B24] Kouzarides T. (2007). Chromatin modifications and their function. *Cell* 128 693–705 10.1016/j.cell.2007.02.005 17320507

[B25] Lasserre J. Chung H.-R. Vingron M. (2013). Finding associations among histone modifications using sparse partial correlation networks. *PLoS Comput. Biol.* 9:e1003168 10.1371/journal.pcbi.1003168 PMC3764007 24039558

[B26] Lessard J. Wu J. I. Ranish J. A. Wan M. Winslow M. M. Staahl B. T. (2007). An essential switch in subunit composition of a chromatin remodeling complex during neural development. *Neuron* 55 201–215 10.1016/j.neuron.2007.06.019 17640523 PMC2674110

[B27] Lindroth A. M. Shultis D. Jasencakova Z. Fuchs J. Johnson L. Schubert D. (2004). Dual histone H3 methylation marks at lysines 9 and 27 required for interaction with CHROMOMETHYLASE3. *EMBO J.* 23 4286–4296 10.1038/sj.emboj.7600430 15457214 PMC524394

[B28] Maeder M. L. Angstman J. F. Richardson M. E. Linder S. J. Cascio V. M. Tsai S. Q. (2013). Targeted DNA demethylation and activation of endogenous genes using programmable TALE-TET1 fusion proteins. *Nat. Biotechnol.* 31 1137–1142 10.1038/nbt.2726 24108092 PMC3858462

[B29] Martin A. M. Pouchnik D. J. Walker J. L. Wyrick J. J. (2004). Redundant roles for histone H3 N-terminal lysine residues in subtelomeric gene repression in *Saccharomyces cerevisiae*. *Genetics* 167 1123–1132 10.1534/genetics.104.026674 15280228 PMC1470950

[B30] Mendenhall E. M. Williamson K. E. Reyon D. Zou J. Y. Ram O. Joung J. K. (2013). Locus-specific editing of histone modifications at endogenous enhancers. *Nat. Biotechnol.* 31 1133–1136 10.1038/nbt.2701 24013198 PMC3858395

[B31] Morinière J. Rousseaux S. Steuerwald U. Soler-López M. Curtet S. Vitte A.-L. (2009). Cooperative binding of two acetylation marks on a histone tail by a single bromodomain. *Nature* 461 664–668 10.1038/nature08397 19794495

[B32] Nguyen U. T. T. Bittova L. Muller M. M. Fierz B. David Y. Houck-Loomis B. (2014). Accelerated chromatin biochemistry using DNA- barcoded nucleosome libraries. *Nat. Methods* 11 834–840 10.1038/nmeth.3022 24997861 PMC4130351

[B33] Pelizzola M. Ecker J. R. (2011). The DNA methylome. *FEBS Lett.* 585 1994–2000 10.1016/j.febslet.2010.10.061 21056564 PMC3129437

[B34] Ram O. Goren A. Amit I. Shoresh N. Yosef N. Ernst J. (2011). Combinatorial patterning of chromatin regulators uncovered by genome-wide location analysis in human cells. *Cell* 147 1628–1639 10.1016/j.cell.2011.09.057 22196736 PMC3312319

[B35] Rando O. J. (2012). Combinatorial complexity in chromatin structure and function: revisiting the histone code. *Curr. Opin. Genet. Dev.* 22 148–155 10.1016/j.gde.2012.02.013 22440480 PMC3345062

[B36] Rivenbark A. G. Stolzenburg S. Beltran A. S. Yuan X. Rots M. G. Strahl B. D. (2012). Epigenetic reprogramming of cancer cells via targeted DNA methylation. *Epigenetics* 7 350–360 10.4161/epi.19507 PMC3368819 22419067

[B37] Rivera C. M. Ren B. (2013). Mapping human epigenomes. *Cell* 155 39–55 10.1016/j.cell.2013.09.011 24074860 PMC3838898

[B38] Rothbart S. B. Strahl B. D. (2014). Interpreting the language of histone and DNA modifications. *Biochim. Biophys. Acta* 1839 627–643 10.1016/j.bbagrm.2014.03.001 24631868 PMC4099259

[B39] Ruthenburg A. J. Li H. Milne T. A. Dewell S. McGinty R. K. Yuen M. (2011). Recognition of a mononucleosomal histone modification pattern by BPTF via multivalent interactions. *Cell* 145 692–706 10.1016/j.cell.2011.03.053 21596426 PMC3135172

[B40] Ruthenburg A. J. Li H. Patel D. J. Allis C. D. (2007). Multivalent engagement of chromatin modifications by linked binding modules. *Nat. Rev. Mol. Cell Biol.* 8 983–994 10.1038/nrm2298 18037899 PMC4690530

[B41] Schreiber S. L. Bernstein B. E. (2002). Signaling network model of chromatin. *Cell* 111 771–778 10.1016/S0092-8674(02)01196-0 12526804

[B42] Strahl B. D. Allis C. D. (2000). The language of covalent histone modifications. *Nature* 403 41–45 10.1038/47412 10638745

[B43] Turner B. M. (2007). Defining an epigenetic code. *Nat. Cell Biol.* 9 2–6 10.1038/ncb0107-2 17199124

[B44] Voigt P. Leroy G. Drury W. J. III Zee B. M. Son J. Beck D. B. (2012). Asymmetrically modified nucleosomes. *Cell* 151 181–193 10.1016/j.cell.2012.09.002 23021224 PMC3498816

[B45] Wang C. Zhang M. Q. Zhang Z. (2013). Computational identification of active enhancers in model organisms. *Genomics Proteomics Bioinformatics* 11 142–150 10.1016/j.gpb.2013.04.002 23685394 PMC4357786

[B46] Wei Y. Yu L. Bowen J. Gorovsky M. A. Allis C. D. (1999). Phosphorylation of histone H3 is required for proper chromosome condensation and segregation. *Cell* 97 99–109 10.1016/S0092-8674(00)80718-7 10199406

[B47] Wu W. Cheng Y. Keller C. A. Ernst J. Kumar S. A. Mishra T. (2011). Dynamics of the epigenetic landscape during erythroid differentiation after GATA1 restoration. *Genome Res.* 21 1659–1671 10.1101/gr.125088.111 21795386 PMC3202283

[B48] Yan Z. Wang Z. Sharova L. Sharov A. A. Ling C. Piao Y. (2008). BAF250B-associated SWI/SNF chromatin-remodeling complex is required to maintain undifferentiated mouse embryonic stem cells. *Stem Cells* 26 1155–1165 10.1634/stemcells.2007-0846 18323406 PMC2409195

[B49] Yu H. Zhu S. Zhou B. Xue H. Han J.-D. J. (2008). Inferring causal relationships among different histone modifications and gene expression. *Genome Res.* 18 1314–1324 10.1101/gr.073080.107 18562678 PMC2493438

[B50] Yuan G.-C. Ma P. Zhong W. Liu J. S. (2006). Statistical assessment of the global regulatory role of histone acetylation in *Saccharomyces cerevisiae*. *Genome Biol.* 7 R70. 10.1186/gb-2006-7-8-r70 PMC1779595 16884527

[B51] Zhou J. Troyanskaya O. G. (2014). Global quantitative modeling of chromatin factor interactions. *PLoS Comput. Biol.* 10:e1003525 10.1371/journal.pcbi.1003525 PMC3967939 24675896

